# Hypercalcemia secondary to excessive self-medication with antacids causing acute pancreatitis: a case report

**DOI:** 10.3325/cmj.2019.60.42

**Published:** 2019-02

**Authors:** Pietro Vassallo, Nikki Green, Edward Courtney

**Affiliations:** 1 University Hospitals Bristol NHS Foundation Trust, Bristol, United Kingdom; 2 Royal United Hospitals Bath NHS Foundation Trust, Bath, United Kingdom

## Abstract

Excessive self-medication with over-the-counter drugs is an issue commonly encountered by health care professionals. It can result in uncommon presentations of life-threatening illnesses. These medications are frequently overlooked by clinicians when taking histories from patients, and their risks are often downplayed. We present the case of a 35-year-old woman with acute pancreatitis secondary to hypercalcemia. This condition occurred due to long-term excessive self-administration of calcium-rich antacid tablets. Her clinical course involved multifactorial rebound hypocalcemia after treatment and multiple complications from the abuse of other non-prescription medications. Acute pancreatitis secondary to antacid-induced milk-alkali syndrome has been minimally reported in the literature. There are no reports describing rebound hypocalcemia as a complication of its treatment or presenting this pathology in the context of multiple over-the-counter drug abuse. This case highlights the importance of taking thorough drug histories, including non-prescription medications, in acute clinical assessments.

Hypercalcemia is a well-recognized etiology of acute pancreatitis, commonly associated with primary hyperparathyroidism (1). However, antacid-induced milk-alkali syndrome as a cause of acute pancreatitis has only been described once (2). Standard care for acute pancreatitis involves aggressive fluid therapy and supportive treatment. This case also included immediate discontinuation of antacids, resulting in severe rebound hypocalcemia requiring replacement. This complication, and the aforementioned pathology in the context of multiple over-the-counter drug abuses, has not been described before.

## Case report

A 35-year-old woman presented to the Royal United Hospital in Bath (United Kingdom) Emergency Department in October 2017 with a three-day history of new onset epigastric pain radiating to the back, associated with vomiting and reduced nutritional intake over several weeks. She had a history of alcohol excess, but her family confirmed she had been abstinent for 3 months before admission. Her medical history included anxiety and depression, which were untreated at the time – her selective serotonin reuptake inhibitor had been stopped several weeks earlier. She also reported chronic back pain, for which she self-medicated using over-the-counter analgesia. She denied ever discussing her self-medication with a health care professional. She had no known gallstone disease and was taking no prescription medications at the time. No further relevant medical, family, or social history was recorded. On examination, she was tachycardic and her abdomen was very tender across the epigastrium.

During the admission clerking, she reported longstanding excessive self-medication with oral antacids and over the counter analgesia. She reported consuming up to 72 calcium carbonate with heavy magnesium carbonate tablets (Rennie Peppermint, Bayer plc, Reading, United Kingdom) per day and 600 mL of sodium alginate with sodium bicarbonate and calcium carbonate liquid (Gaviscon Original Aniseed Relief, Reckitt Benckiser Healthcare Limited, Hull, United Kingdom) per week over the past 8 months to tackle reflux symptoms. Both these medications are rich in calcium (3,4). She also reported taking up to 6 g of ibuprofen and 7.5 g of paracetamol per day for her back pain – respectively 2.5 and 1.9 times the maximum recommended daily doses for adults according to the British National Formulary (5).

Admission blood tests showed raised white cells (19.2 × 10^9^/L), C-reactive protein (118 mg/L), and amylase (2121 U/L). Corrected calcium was raised at 3.82 mmol/L. Venous blood gas highlighted a metabolic alkalosis, with pH 7.451 and raised base excess (+3.8 mEq) and bicarbonate (28.1 mEq/L). Deranged liver function and clotting were also found. Ultrasound scan of the abdomen detected no gallstones. Due to raised calcium, she was treated for hypercalcemic acute pancreatitis secondary to excessive antacid administration (Modified Glasgow Score: 2). This was described as acute pancreatitis secondary to milk-alkali syndrome with preserved renal function (6). She underwent aggressive intravenous and oral fluid resuscitation to lower calcium and replace electrolytes. She also received N-acetylcysteine for accidental paracetamol overdose, and vitamin K for deranged clotting. Her adjusted calcium dropped steadily throughout hospitalization, reaching its trough on day 6 (1.70 mmol/L) and resulting in severe rebound hypocalcemia. This was attributable to sudden discontinuation of antacids and calcium sequestration due to acute pancreatitis (7). Oral and intravenous replacement restored normal calcium levels (adjusted calcium 2.29 mmol/L on discharge on day 10).

In addition, on day 2 after admission her hemoglobin dropped significantly (90 g/L to 65 g/L). Due to the history of excessive non-steroidal anti-inflammatory drug use, urgent gastroscopy was carried out to exclude peptic ulcer bleeding. Two non-bleeding 10 mm gastric ulcers were found at the incisura and pylorus. Rapid urease test was negative, associating the ulcers with non-steroidal anti-inflammatory drug-induced gastric irritation. Her drug chart showed that she had received over 5.5 L of intravenous fluids in 24 hours, before the hemoglobin drop. The hemoglobin drop was attributed to hemodilution – this recovered steadily after 2 units of packed red blood cells. High dose proton pump inhibitor was also commenced to treat the ulcers.

**Figure 1:**
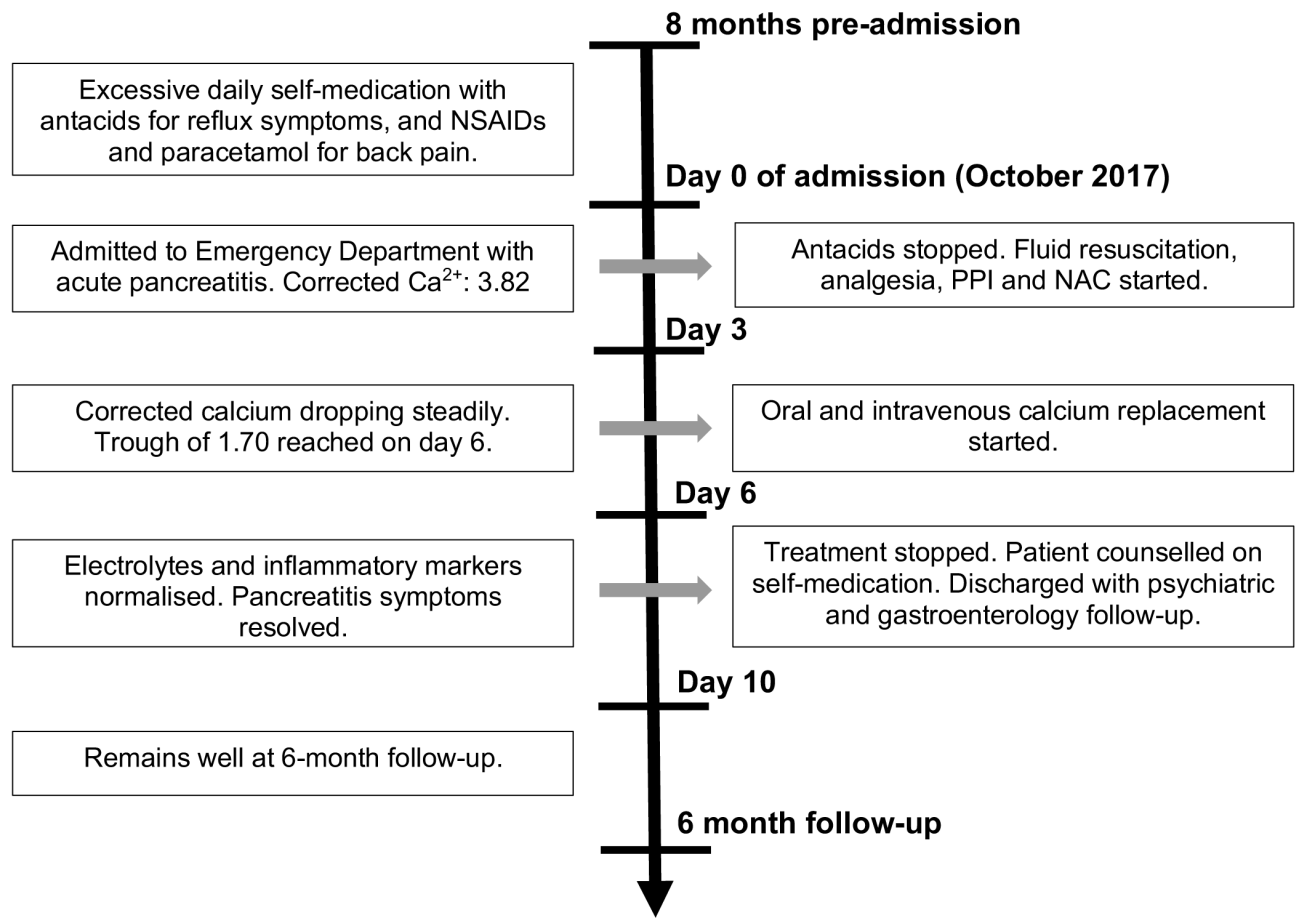
Timeline of events and treatment interventions. NSAIDs – nonsteroidal anti-inflammatory drugs; PPI – proton pump inhibitors; NAC – N-acetylcysteine.

Ten days post-admission, her electrolytes normalized, inflammatory markers improved, and pancreatitis symptoms resolved. She received counselling regarding excessive self-medication and reported to understand its serious consequences at the time. She was advised to seek further support in relation to her self-medication and mood, and follow-up was arranged to see community mental health services. The patient was discharged home with gastroenterology follow-up. Six months later she remained asymptomatic, with normal electrolytes and no further evidence of excessive self-medication. A timeline of events can be seen in Figure 1.

## Discussion

We described a rare complication of milk-alkali syndrome. Milk-alkali syndrome consists of hypercalcemia, renal failure, and metabolic alkalosis secondary to the consumption of excessive calcium and alkali (6). Excessive calcium ingestion is usually considered as more than 4-5 g of calcium carbonate per day (8,9). Rennie Peppermint tablets contain 680 mg calcium carbonate per tablet, while Gaviscon Original Aniseed Relief liquid contains 80 mg calcium carbonate per 5 mL (3,4). Our patient was consuming considerably more than 4-5 g of calcium carbonate per day in the form of antacids, resulting in serum hypercalcemia.

Hypercalcemia is a widely recognized cause of acute pancreatitis (1,10). However, only one previous report describes excessive antacid administration as the etiology (2). Antacids’ flavored palatability and unregulated purchase makes them prone to excessive self-administration. However, as they are not advertised as calcium supplements, health professionals often overlook their high calcium content. This can result in the missed recognition of antacids as etiological factors in acute pancreatitis, thus delaying diagnosis and increasing inappropriate investigations and interventions for these patients.

Our patient also suffered rebound hypocalcemia after treatment. This was secondary to interruption of the antacids, intravenous fluid resuscitation, and calcium sequestration secondary to acute pancreatitis itself (7). This highlights the importance of careful calcium monitoring during care, as well as considering early careful calcium replacement once the causative agent is discontinued.

Overall, this case highlights the multifactorial complications of excessive self-medication with over-the-counter drugs. These can be life-threatening illnesses and can result from apparently safe medications like antacids. Clinicians should also recognize that patients can abuse several types of medications, which can result in complex clinical pictures and unusual presentations of common pathologies.
